# Chinese Herb *Astragalus membranaceus* Enhances Recovery of Hemorrhagic Stroke: Double-Blind, Placebo-Controlled, Randomized Study

**DOI:** 10.1155/2012/708452

**Published:** 2012-03-12

**Authors:** Chun-Chung Chen, Han-Chung Lee, Ju-Hsin Chang, Shuang-Shuang Chen, Tsai-Chung Li, Chang-Hai Tsai, Der-Yang Cho, Ching-Liang Hsieh

**Affiliations:** ^1^Department of Neurosurgery, China Medical University Hospital, Taichung 40402, Taiwan; ^2^Institute of Integrated Medicine, College of Chinese Medicine, China Medical University, Taichung 40402, Taiwan; ^3^School of Medicine, College of Medicine, China Medical University, Taichung 40402, Taiwan; ^4^Department of Anesthesiology, China Medical University Hospital, Taichung 40402, Taiwan; ^5^Graduate Institute of Biostatistics, College of Public Health, China Medical University, Taichung 40402, Taiwan; ^6^Division of Pediatric Neurology, Department of Pediatrics, China Medical University, Taichung 40402, Taiwan; ^7^Graduate Institute of Acupuncture Science, College of Chinese Medicine, China Medical University, Taichung 40402, Taiwan; ^8^Acupuncture Research Center, China Medical University, Taichung 40402, Taiwan; ^9^Department of Chinese Medicine, China Medical University Hospital, Taichung 40402, Taiwan

## Abstract

We tested the effect of *Astragalus membranaceus* (AM) on acute hemorrhagic stroke. Seventy-eight patients were randomly assigned to Group A (3 g of AM three times/day for 14 days); or Group B (3 g of placebo herb). A total of 68 patients (Group A 36, Group B 32) completed the trial. The increase of functional independence measure scale score between baseline and week 4 was 24.53 ± 23.40, and between baseline and week 12 was 34.69 ± 28.89, in the Group A was greater than 11.97 ± 11.48 and 23.94 ± 14.8 in the Group B (both *P*≦0.05). The increase of Glasgow outcome scale score between baseline and week 12 was 0.75 ± 0.77 in the Group A was greater than 0.41 ± 0.50 in the Group B (*P* < 0.05). The results are preliminary and need a larger study to assess the efficacy of AM after stroke.

## 1. Introduction

Intracerebral hemorrhage (ICH) is a subtype of stroke with high morbidity and mortality, accounting for approximately 15% of all deaths from stroke [1]. Many patients make only a partial or poor recovery, and 36% of acute hemorrhagic stroke patients remain moderately to severely disable at discharge [2]. Therefore, treatments to enhance their recovery are necessary. Clinical research performed in China based on traditional Chinese medicine (TCM) may reveal new possibilities for the treatment of strokes. However, these treatments have limited acceptability outside China because of the Western medical world's unfamiliarity with the TCM concept of stroke, which is relatively different from the Western view.

Pharmacological studies have demonstrated that several TCM herbs possess antioxidant, antiinflammatory, and antiglutamate properties [3]. Such herbs can dilate blood vessels, suppress platelet aggregation, protect against ischemic reperfusion injury, and enhance the tolerance of ischemic tissue to hypoxia [4]. *Astragalus membranaceus* (AM) is a traditional Chinese herb that has been used extensively in China as a drug to facilitate recovery after a stroke. Clinical studies performed in China have shown that AM enhances stroke patients' recovery from their neurological disability and improves functional outcome [5, 6]. However, these trials did not comply with the International Conference on Harmonization and Good Clinical Practice guidelines, and they used positive controls. Furthermore, the outcome measures were not the standard scales used in modern stroke trials. One study [5, 6] suggested that the effectiveness of AM in improving stroke recovery may be related to the herb's role in reducing the area of cerebral infarction area, and its antioxidative qualities. Because no previous studies had researched the use of AM with hemorrhagic stroke patients, we investigated whether AM can enhance the functional recovery of hemorrhagic stroke patients. Our study was conducted in accordance with the International Conference on Harmonization and Good Clinical Practice guidelines. The objective of this research was to obtain pilot data to support the design of a larger, controlled trial in the future.

 Ganglionic (putamen, caudate, and thalamus) hemorrhages are the most common forms of ICH, followed by lobar hemorrhages, and then those of the cerebellum or pons [1]. Location is important for outcome (pontine hemorrhages result in higher mortality), potential surgical intervention, and underlying cause. To evaluate the efficacy of AM, we chose patients who had the same location of ICH, namely, putaminal ICH, to minimize bias. 

## 2. Materials and Methods 

### 2.1. Subjects

Patients were recruited from China Medical University Hospital's neurosurgery and emergency departments between January 1, 2008 and December 21, 2010. All patients were recruited within 24 hours after the onset of hemorrhagic stroke. The experimental procedures complied with the ethical principles dictated in the Declaration of Helsinki, and the protocol of the trial was approved by the institutional review board of China Medical University Hospital, Taichung City, Taiwan (IRB: DMR 96-IRB-126). The trial was conducted according to the International Conference on Harmonization and Good Clinical Practice guidelines. Patients gave their informed consent to participate. 

The criteria for including patients in the study were as follows: (1) female or male; (2) aged between 30 and 75 years; (3) randomized allocation to a study group within 24 hours of hemorrhagic stroke onset; (4) this was the patient's first hemorrhagic stroke, and the location of hemorrhage was the putamen; (5) treatment may or may not have been included surgery; (6) the subject or their legal representative gave written informed consent to participate. The exclusion criteria were as follows: (1) recent thrombolysis treatment; (2) history of previous stroke; (3) full-dose or long-term anti-coagulation therapy; (4) hemorrhagic stroke but the location was not putamen; (5) coexisting systemic diseases such as terminal cancer, renal failure, liver cirrhosis, severe dementia, or psychosis; (6) participation in another clinical trial within the last three months; and (7) pregnancy or lactation. 

### 2.2. Preparation of AM

The AM was purchased from Shansi Province, China. The origin of the herb was authenticated, and the material was examined for microorganisms, heavy metals, and pesticide according to the accepted standards (good manufacturing practice or GMP) of Taiwan. The AM was found to be of good quality, and the crude AM had been extracted by Sun Ten Pharmaceutical Co. Ltd., Taiwan. The AM was extracted at a rate of 3.0 g from every 3.3 g of crude AM. The freeze-dried extracts of AM were verified by high-performance liquid chromatography using *Astragaloside *IV (Biotic Chemical Co. Ltd., China, Shanghai) as an active component of AM. Finally, each 3 g extract was sealed in an aluminum foil sachet. The placebos were also made by Sum Ten Pharmaceutical Co. Ltd. and were manufactured from starch and sealed inside identical foil sachets. 

### 2.3. Design and Sample Size

The present study was a single-center, double-blind, placebo-controlled, randomized phase II pilot study. The sample size was calculated according to our hypothesis that AM can increase a patient's score on ten dimensions of the functional independence measure Scale (FIM), and also on the Barthel Index (BI). We further hypothesized that the increase in score would be evident when comparing scores at baseline (the week of the stroke onset) and week 4, and again at week 12. To achieve a statistical power of 90%, the sample size would need to be 46 patients, and thus, 23 patients in each group. If the rate of followup was 0.8, we would need to recruit 58 patients (46 ÷ 0.8 = 58). 

### 2.4. Randomization and Grouping

Random numbers were generated by computer, using block randomization with a block size of 2 or 4. The pregenerated random numbers were placed in sealed envelopes, and a serial number was assigned to each envelope according to the sequence of allocation of the randomized number. Each envelope was then opened sequentially, according to the admission sequence of subjects at the study center. The number inside the envelope determined which group the subject was allocated to. 

Subjects who were randomly assigned to Group A received oral or nasopharyngeal administration of AM at a rate of 3 g three times per day for 14 days continuously starting within 24 hours after stroke onset except standard treatment that was according to Guidelines for the Management of spontaneous intracerebral hemorrhage in adults (2007 update of American Stroke Association [7]). Patients who were randomly assigned to Group B received the placebo treatment, according to exactly the same schedule as for Group A. Subjects as well as investigators and pharmacists were blinded to the patients' allocation to each group. The password for the randomization envelope for each subject was known only by a designated researcher. 

### 2.5. Outcome Measures

The primary outcome measures were the differences in patients' scores on several clinical scales, between baseline (within  7 ± 1  days after the onset of stroke) and week 4 (28 ± 4 days), and between at baseline and week 12 (84 ± 10 days). The scales we used FIM, BI, Glasgow Outcome Scale (GOS), and Modified Rankin Scale (MRS). The scores of FIM, BI, GOS, and MRS were assessed by an experienced research nurse. 

The secondary outcome measures were as follows:

1. inflammatory index, which included the levels of C-reactive protein (CRP) and erythrocyte sediment rate (ESR) for venous blood; these were measured at baseline (prior to the first AM dose), and again on the fourth and seventh day of admission;
2. computer tomography (CT) examination, which was done at baseline and on the fourth and seventh days of admission. The volume of hematoma was calculated the simplified equation 1/2  *A* × *B* × *C*, where *A* is the maximum width measured, *B* is the length, and *C* is the height [8]. The ratio of brain edema was calculated by CT (volume of edema divided by blood clot volume).

### 2.6. Statistical Analysis

Baseline variables were compared using a two-group *t*-test for continuous variables (e.g., age) and chi-squared (*χ* ^2^) test for categorical variables (e.g., gender). Intention-to-treat analysis was used. For efficacy variables, comparisons were made between the two groups at baseline and weeks 4 and 12, respectively. The two-sample *t-*test was used separately for each comparison. To allow for the possibility of nonnormal distribution, the non-parametric Mann-Whitney test was performed. All analyses were performed using SAS version 9.2 (SAS Institute Inc., Cary, NC). A *P-*value of ≦0.05 was considered statistically significant. 

## 3. Results

### 3.1. Baseline Characteristics of Demographic Data

Eighty subjects were recruited in this study, but one patient was over the age of 75 and another patient declined to participate, leaving a total of 78 patients randomized to assign either the Group A or the Group B. 

**Figure 1:**
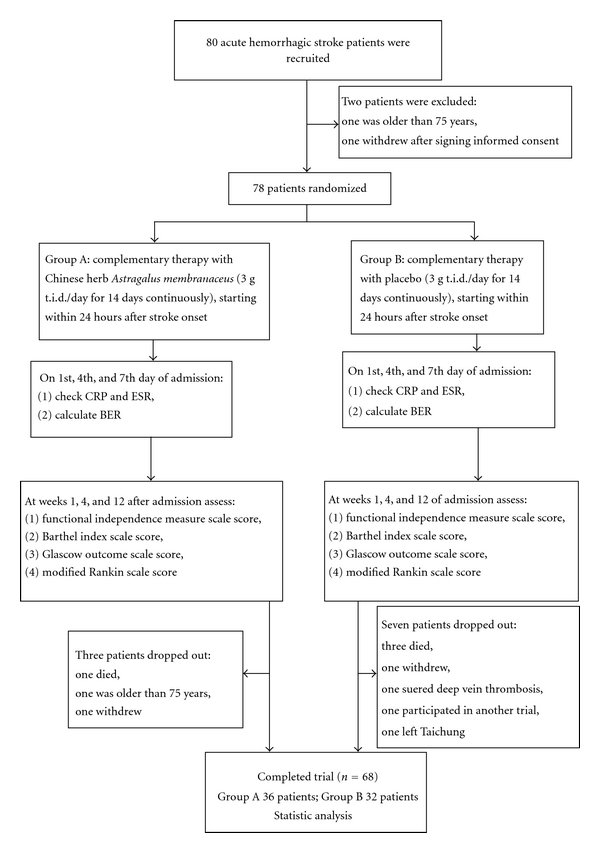
Flowchart.

A total of 68 patients completed the trial, 36 patients in Group A and 32 patients in Group B. The baseline characteristics of Group A and Group B patients regarding gender, age, body temperature (BT), craniotomy, and so forth were summarized in Table 1. Three patients dropped out of Group A (one was older than 75, one withdrew, one died); and seven patients dropped out of Group B (one withdrew, one attended another program, one suffered deep vein thrombosis, one left Taichung, and three died) see Figure 1. 

### 3.2. Primary Outcome Measures

**Figure 2:**
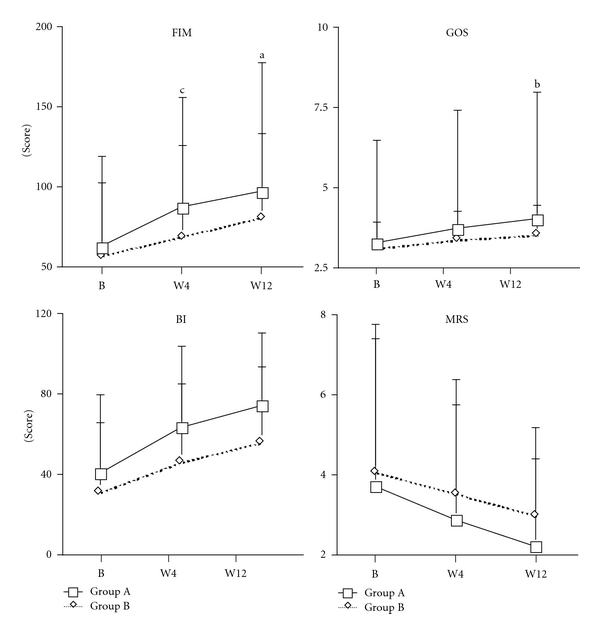
Effect of *Astragalus membranaceus* on primary outcome measures in acute hemorrhagic stroke patients. The increase of FIM scale scores in the group A was greater than in the group B in week 4 and in week 12 in acute hemorrhagic stroke patients. Group A: complementary therapy with *Astragalus membranaceus*; Group B: complementary therapy with placebo; FIM: functional independence measure; BI: Barthel index; GOS: Glasgow outcome scale; MRS: Modified Rankin scale; B: baseline (week 1); W4: week 4; W12: week 12; (a) *P* = 0.05; (b) *P* < 0.05; (c) *P*≦0.01 compared to the increase of Group B.

The FIM scale score at baseline was similar in the two groups, namely, 62.42 ± 40.32 for Group A and 56.97 ± 35.82 for Group B (*P* > 0.05; Figure 2). The increase of FIM scale score between baseline and week 4 was 24.53 ± 23.40, and between baseline and week 12, it was 34.69 ± 28.89: in the Group A was greater than 11.97 ± 11.48  and 23.94 ± 14.8 in the Group B (both *P*≦0.05; Figure 2). 

**Figure 3:**
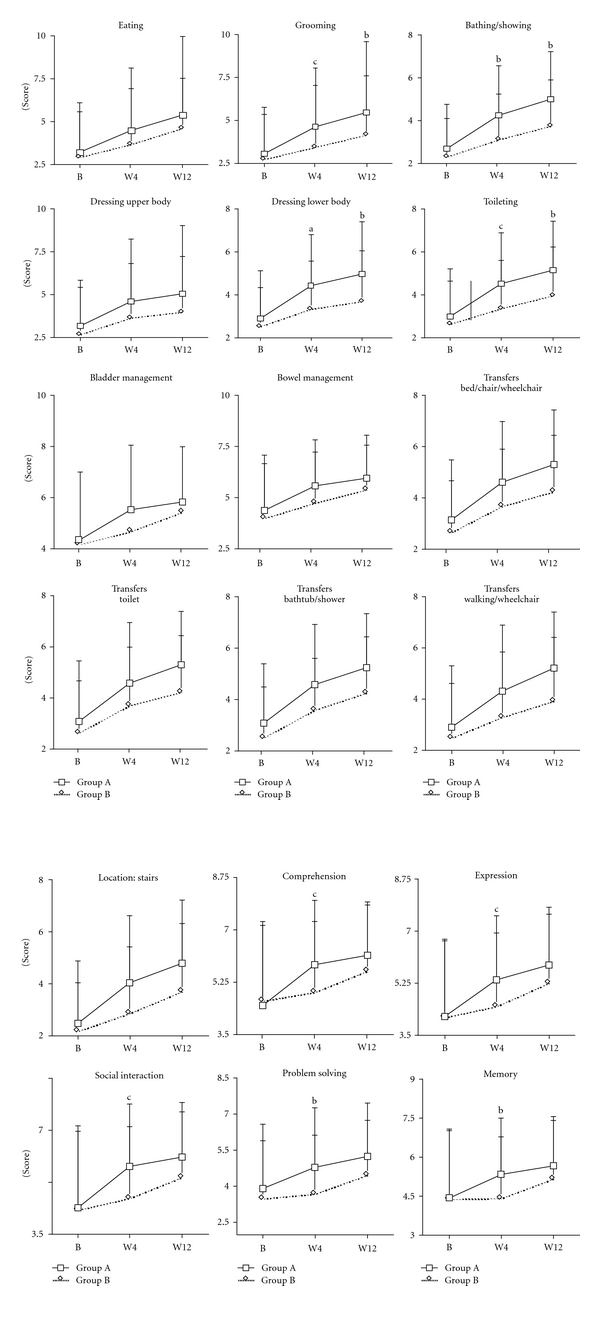
Effect of *Astragalus membranaceus* on sub-scale of FIM scores in acute hemorrhagic stroke patients. The increase of FIM subscale scores in grooming, bathing/showing, dressing lower body and toileting was greater in the group A than in the group B in week 4 and in week 12 in acute hemorrhagic stroke patients. Group A: complementary therapy with *Astragalus membranaceus*; Group B: complementary therapy with placebo; B: baseline (week 1); W4: week 4; W12: week 12; (a) *P* = 0.05; (b) *P* < 0.05; (c) *P*≦0.01 compared to the increase of Group B.

 No statistically significant difference was found between the two groups' FIM subscales scores, at baseline, for the following subscales: eating, dressing upper body, bladder management, bowel management, transfers (bed/chair/wheelchair), transfers (toilet), transfers (bathtub/shower), transfers (walking/wheelchair) and locomotion (stairs), all *P* > 0.05; Figure 3. 

FIM subscale scores at baseline, and between baseline and week 12 were similar between Group A and Group B (all *P* > 0.05; Figure 3), whereas Group A achieved a significantly greater difference than Group B between baseline and week 4 for the subscales: comprehension (*P* < 0.05), expression (*P* < 0.05), social interaction (*P*≦0.01), problem solving (*P* < 0.05), and memory (*P* < 0.05) (Figure 3). 

 The findings shown in Figure 3 indicate that Group A achieved significantly greater score changes than did Group B in several FIM domains. Group A achieved a significantly greater difference than Group B for the subscale “grooming,” between baseline and week 4 (*P*≦0.01), and between baseline and week 12 (*P* < 0.05). Group A also achieved a significantly greater difference than Group B for the subscale “bathing/showering,” between baseline and week 4 (*P* < 0.05), and between baseline and week 12 (*P* < 0.05). Group A score changes for the subscale “dressing lower body” were also significantly higher than those of Group B at both time intervals (baseline to week 4, *P* = 0.05); baseline to week 12 (*P* < 0.05). Group A score changes for the subscale “toileting” were significantly higher than those of Group B at both time intervals (baseline to week 4, *P*≦0.01; baseline to week 12, *P* < 0.05). The score differences for other subscales are summarized in Figure 3. 

As shown in Figure 2, the two groups had similar BI scores at baseline (39.86 ± 38.87 in Group A, and 30.94 ± 33.92 in Group B; *P* > 0.05). There was also no statistically significant difference between Group A and B changes in scores from baseline to week 4, or from baseline to week 12 (both *P* > 0.05). 

 The GOS score at baseline was 3.25 ± 0.91 for Group A, and 3.13 ± 0.83 for Group B, with the difference between the groups not being significant (*P* > 0.05; Figure 2). The difference in GOS scores between baseline and week 4 was 0.47 ± 0.61 for Group A, and 0.25 ± 0.44 for Group B, which was not significant (*P* > 0.05; Figure 2). The difference in GOS score between baseline and week 12 was 0.75 ± 0.77 for Group A, and 0.41 ± 0.50 for Group B, with the difference between the groups being significant (*P* < 0.05; Figure 2). 

The MRS score at baseline was 3.69 ± 1.51 for Group A, and 4.06 ± 1.16 for Group B, with the difference between groups not being significant (*P* > 0.05; Figure 2). For Group A, the difference in MRS score between baseline and week 4 was −0.83 ± 0.94, and between baseline and week 12 it was −1.50 ± 1.23. These results were not significantly different from those of Group B, namely, −0.56 ± 0.80 and −1.09 ± 1.96, respectively, both *P* > 0.05; Figure 2. 

### 3.3. Secondary Outcome Measure

**Figure 4:**
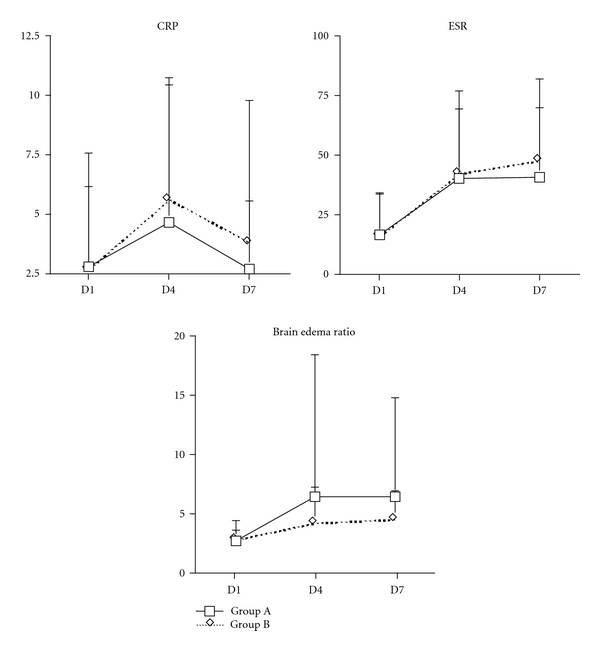
Effect of *Astragalus membranaceus* on secondary outcome measures in acute hemorrhagic stroke patients. The increase of C-reactive protein (CRP) levels and erythrocyte sediment rate (ESR), and brain edema ratio in the group A was similar to in the group B in day 4 and in day 7 in acute hemorrhagic stroke patients. Group A: complementary therapy with *Astragalus membranaceus*; Group B: complementary therapy with placebo; D1: baseline, prior to administration of *Astragalus membranaceus*; D4: fourth day of admission; D7: seventh day of admission.

As shown in Figure 4, the level of CRP on day 1 was 2.80 ± 4.71 for Group A, which was not significantly different from the results for Group B, namely, 2.69 ± 3.44 (*P* > 0.05). For Group A, the change in CRP between days 1 and 4 was 1.82 ± 4.44, and between days 1 and 7 it was −0.13 ± 3.98. These results were not significantly different from those of Group B, namely, 2.90 ± 4.24 and 1.08 ± 6.87, respectively (both *P* > 0.05) for the intergroup differences at the two time periods, respectively. 

The level of ESR on day 1 was 16.28 ± 17.11 for Group A, similar to 15.72 ± 18.11 for Group B (*P* > 0.05; Figure 4). The change in ESR for Group A was 23.28 ± 21.30 between days 1 and 4, and 24.06 ± 21.24 between days 1 and 7. These results were similar to those of Group B, namely, 25.88 ± 27.47 and 31.78 ± 28.27, respectively (both *P* > 0.05; Figure 4). 

The level of BER on day 1 was 2.61 ± 0.92 for Group A, similar to 2.72 ± 1.68 for Group B (*P* > 0.05; Figure 4). The change in BER for Group A was 3.70 ± 11.93 between days 1 and 4, and 3.73 ± 8.41 between days 1 and 7. These results were similar to those of Group B, namely, 1.39 ± 2.82 and 1.76 ± 2.34, respectively (both *P* > 0.05; Figure 4). 

### 3.4. Adverse Effects

A total of 13 severe adverse events (SAE) occurred in 10 patients (5 events in 4 patients of Group A; 8 events and 6 patients in Group B). The SAEs included prolonged admission (1 patient), second operation (2 patients), respiratory failure (1 patient), pneumonia (1 patient), ventriculoperitoneal shunt operation (1 patient), uterine myoma (1 patient), urinary tract infection (1 patient), deep venous thrombosis (1 patient), and four patients' deaths (1 patient in Group A, and 3 in Group B). More minor adverse events such as dizziness (13 patients), skin rash (2 patients), and fever (22 patients) were also noted. The SAEs were considered not to be related to the medication under study. The deaths were considered to be due to rebleeding of the intracerebral hematoma, which caused increased intracranial pressure; this was not related to the study medications. 

## 4. Discussion

Our results indicated that the FIM and GOS scores of Group A (patients treated with complementary therapy AM) were similar to those of Group B (placebo) at baseline. The increase in FIM scores was greater for Group A at week 4 and again at week 12, relative to Group B. The increase in GOS scores was greater for Group A than Group B at week 12. The score changes for BI and MRS were not significantly different at week 4 and at week 12 compared to the first week after stroke onset. 

Therefore, we suggest that AM provides an advantage for acute hemorrhagic stroke patients, if treatment with AM is started within 24 hours of stroke onset. In particular, we found that AM therapy enhanced patients' functional recovery for grooming, bathing, showering, dressing the lower body, and toileting. Additionally, the results show an excellent safety profile for treatment with AM; overall, the treatment was well-tolerated and none of the observed SAEs were considered drug related.

The pathological changes because of brain injury after ICH include hematoma expansion, midline shift, and brain edema. Enlargement of the hematoma after ictus contributes to midline shift and accelerates neurological deterioration [9–11]. Perihematomal brain edema develops immediately after an ICH and peaks several days later [12, 13]. In humans, perihematomal edema develops within 3 h of symptom onset and peaks between 10 and 20 days after ictus [14, 15]. The formation of edema after ICH increases intracranial pressure and can result in herniation [16, 17]. Several studies have shown that the degree of brain edema around the hematoma is associated with outcome, with worse edema being associated with poorer outcomes [11, 18]. There are several phases of edema formation after ICH. The early phase (first few hours) involves hydrostatic pressure and clot retraction, with movement of serum from the clot into the surrounding tissue [19]. The second phase (first two days) is related to the coagulation cascade and thrombin production; and the third phase is related to erythrocyte lyses and haemoglobin toxicity. Therefore, the mechanism of edema includes hematoma, oxidation, and inflammation. 

Therapy with AM has the effect of antioxidation and anti-inflammation, which indicates that AM can decrease edema and improve the patient's prognosis [20, 21]. Our results showed that AM therapy improved functional outcomes at week 4 and at week 12 after hemorrhagic stroke onset, which may be due to AM's properties of anti-inflammation and antioxidation, which would decrease brain edema. 

A growing body of evidence suggests that inflammation after both ischemic stroke and ICH or higher brain edema is predominantly deleterious. In human stroke, prestroke infection is associated with worse outcome, as recently reported in a review by McColl and colleagues [22]. High levels of CRP, which is an acute-phase protein released by the liver in response to IL-6, are linked to worse outcome following stroke [23], and acute prestroke administration of human CRP is deleterious in experimental models [24]. It may be that CRP enhances complement-mediated neutrophil chemotaxis and degranulation. Inflammation aggravates hemorrhagic brain injury. An inflammatory response in the surrounding brain occurs soon after ICH and peaks several days later in human beings and in animals [25, 26]. In addition to CRP, another indicator of inflammation is ESR. 

Our results leave one question unanswered. That is, why did FIM scores increase more for Group A than Group B at weeks 4 and 12, and GOS scores also increased more in Group A at week 12 relative to Group B, whereas the groups did not show any significant differences regarding changes in CRP, ESR, and BER? In response to this question, we suggest that the effect of AM effect gradually increases, with the greatest effect occurring after seven days. Our study design was such that we stopped measuring CRP and ESR and performing CT scans after the seventh day; we measured on days 1, 4, and 7 after admission. This possible explanation requires further study. 

 One study, which reviewed data from 586 patients with ICH seen at 30 different medical centers, reported that hemorrhagic stroke mortality at 3 months was 34% [27]. Ganglionic (putamen, caudate, and thalamus) hemorrhages are the most common forms of ICH, followed by lobar, and then cerebellar or pontine [1]. Location is important for outcome (pontine hemorrhages result in higher mortality), potential surgical intervention, and underlying cause. Our results indicated that the mortality of putaminal ICH is approximately 5 to 6%, which is lower than for average ICH. Therefore, putaminal ICH is associated with a better outcome than other locations of ICH. 

 Our study was subject to some limitations. The sample size of 68 subjects was insufficient to draw any firm conclusions on the efficacy of the treatment. The study itself was an exploratory analysis, with the objective of generating hypotheses for future, larger trials. In addition, our data analysis did not consider the inflation of type I error due to multiple comparisons. All observed results were not statistically significant if Bonferroni correction for multiple comparisons was made. Although testing multiple response variables with Bonferroni correction is technically correct, it is seldom used by most of clinical trials in the literature. However, trends were observed, and our results provided some estimates for sample sizes, which would be required to achieve statistical significance in future studies. The duration of the treatment and of the study itself was shorter than that of other trials assessing the efficacy of AM after stroke. This suggests that a longer trial period could also be an important criterion for subsequent protocols. 

In conclusion, the findings of our study are preliminary, and a larger study to assess the efficacy of AM after stroke is needed. 

## Figures and Tables

**Table 1:** Demographic characteristics at baseline.

	Group A	Group B	*P*-value*
	(*n* = 36)	(*n* = 32)	

Gender			0.74
Female	10 (27.78)	11 (34.38)	
Male	26 (72.22)	21 (65.63)	
Age (yrs)	56.08 ± 10.15	54.94 ± 12.77	0.68
Age			0.78
<65	28 (77.78)	23 (71.88)	
≥65	8 (22.22)	9 (28.13)	
BT	36.85 ± 0.71	37.36 ± 3.58	0.43
Hematoma volume	27.94 ± 40.22	28.61 ± 28.25	0.94
Craniotomy			1.00
No	25 (69.44)	22 (68.75)	
Yes	11 (30.56)	10 (31.25)	
Midline deviation			1.00
No	30 (85.71)	28 (87.50)	
Yes	5 (14.29)	4 (12.50)	
GPT	30.70 ± 22.59	35.39 ± 32.19	0.57
GOT	48.54 ± 70.97	35.68 ± 20.16	0.40
BUN	12.72 ± 4.20	15.47 ± 11.60	0.21
Creatinine	2.26 ± 8.19	0.95 ± 0.50	0.34
PT	11.11 ± 0.65	11.20 ± 0.71	0.57
PTT	29.63 ± 2.73	30.00 ± 3.50	0.62
Hb	14.31 ± 2.01	15.04 ± 1.45	0.10
Plt	210.78 ± 55.04	216.00 ± 49.90	0.68
Hypertension			0.23
No	15 (41.67)	8 (25.00)	
Yes	21 (58.33)	24 (75.00)	
Hyperlipidemia			0.47
No	36 (100.00)	31 (96.88)	
Yes	0 (0.00)	1 (3.13)	
Hyperuricemia			1.00
No	35 (97.22)	32 (100.00)	
Yes	1 (2.78)	0 (0.00)	
DM			0.14
No	34 (94.44)	26 (81.25)	
Yes	2 (5.56)	6 (18.75)	
ICU			0.46
No	3 (8.33)	5 (15.63)	
Yes	33 (91.67)	27 (84.38)	
Angina			0.47
No	36 (100.00)	31 (96.88)	
Yes	0 (0.00)	1 (3.13)	
Anemia			1.00
No	35 (97.22)	31 (96.88)	
Yes	1 (2.78)	1 (3.13)	
DU bleeding			1.00
No	35 (97.22)	32 (100.00)	
Yes	1 (2.78)	0 (0.00)	
COPD			0.47
No	36 (100.00)	31 (96.88)	
Yes	0 (0.00)	1 (3.13)	
Arrhythmia			—
No	36 (100.00)	32 (100.00)	
Yes	0 (0.00)	0 (0.00)	
RCC			1.00
No	35 (97.22)	32 (100.00)	
Yes	1 (2.78)	0 (0.00)	
TB			1.00
No	35 (97.22)	32 (100.00)	
Yes	1 (2.78)	0 (0.00)	
Hypercholesterolemia			0.47
No	36 (100.00)	31 (96.88)	
Yes	0 (0.00)	1 (3.13)	

() represent %; Group A: complementary therapy with *Astragalus membranaceus*; Group B: complementary therapy with placebo; BT: body temperature; craniotomy: craniotomy treatment with craniotomy; midline deviation: midline of brain deviated to right hemisphere or left hemisphere; GPT: glutamic pyruvic transaminase; GOT: glutamic oxaloacetic transaminase; BUN: blood urea nitrogen; PT: prothrombin time; PTT: partial thromboplastin; Hb: hemoglobin; Plt: platelets; DM: diabetes mellitus; ICU: intensive care unit; Angina: angina pectoris; DU bleeding: duodenal ulcer bleeding; COPD: chronic obstructive pulmonary disease: RCC: coronary care unit; TB: pulmonary tuberculosis. *t-test for independent groups was used for used the continuous data; chi-square test or fisher's exact test for categorical data.
